# Selection for brain size impairs innate, but not adaptive immune responses

**DOI:** 10.1098/rspb.2015.2857

**Published:** 2016-03-16

**Authors:** Alexander Kotrschal, Niclas Kolm, Dustin J. Penn

**Affiliations:** 1 Department of Zoology/Ethology, Stockholm University, Svante Arrhenius väg 18B, Stockholm 10691, Sweden; 2 Konrad Lorenz Institute of Ethology, Department of Integrative Biology and Evolution, University of Veterinary Medicine, Vienna, Savoyenstraße 1a, Vienna 1160, Austria

**Keywords:** brain size, immune response, trade-off

## Abstract

Both the brain and the immune system are energetically demanding organs, and when natural selection favours increased investment into one, then the size or performance of the other should be reduced. While comparative analyses have attempted to test this potential evolutionary trade-off, the results remain inconclusive. To test this hypothesis, we compared the tissue graft rejection (an assay for measuring innate and acquired immune responses) in guppies (*Poecilia reticulata*) artificially selected for large and small relative brain size. Individual scales were transplanted between pairs of fish, creating reciprocal allografts, and the rejection reaction was scored over 8 days (before acquired immunity develops). Acquired immune responses were tested two weeks later, when the same pairs of fish received a second set of allografts and were scored again. Compared with large-brained animals, small-brained animals of both sexes mounted a significantly stronger rejection response to the first allograft. The rejection response to the second set of allografts did not differ between large- and small-brained fish. Our results show that selection for large brain size reduced innate immune responses to an allograft, which supports the hypothesis that there is a selective trade-off between investing into brain size and innate immunity.

## 1. Introduction

Organisms do not have unlimited resources and therefore when natural selection favours increased investment into one trait, there are fewer resources to invest into other traits [1]. This limitation is the basis for life-history trade-offs, and can potentially restrict or bias evolutionary pathways [2–5]. Brain size varies dramatically among species (over four orders of magnitude among vertebrates) [6], and the focus of much research is to explain the evolution of brain size and the selective trade-offs of large brains (e.g. [7–11]). There is evidence for selective benefits of larger brains [12], which are likely to arise from improved cognitive abilities [11,13–15], but then why does brain size vary so much? Why do not all animals have large brains? It has long been assumed that large brain size imposes selective trade-offs with other traits. Identifying these selective trade-offs is vital for understanding brain evolution.

Uncovering functional trade-offs is difficult, and negative statistical correlations between traits are generally used as indicators of such trade-offs [2]. Several traits show negative associations with brain size on the interspecific level, such as gut size in primates [16], birds [17] and cichlid fishes [18], fat storage in mammals [19] and a pipefish [20], and testes mass in bats [21]. For the evolution of vertebrate brain size, those negative associations are usually interpreted to support the hypothesis that investment into one ‘expensive’ tissue is traded off against investment into other energetically costly traits [16]. But the precise mechanisms underlying trade-offs between brain size and other traits are still unclear. For example, the negative association between brain and fat tissue in mammals may arise due to biochemical constraints of mammalian energy generation rather than a direct energetic trade-off where an increased investment into brain tissue may lead to decreased fat deposition [22]. Also, a negative association between brain and testis mass is not apparent in all mammals [23]. Further, since comparative analyses are correlational, the hypothesis that brain size has selective trade-offs with other traits requires experimental testing. Artificial selection over multiple generations provides one of the best approaches for evaluating functional trade-offs between the trait that has been selected and other trait(s) [24,25]. For example, a recent series of experiments with guppies (*Poecilia reticulata*) artificially selected for large and small brains confirmed a trade-off between brain size and gut size, as large-brained animals also evolved smaller guts [11]. Our aim here was to use fish from these selection lines to test whether selection for increased brain size similarly reduced the function of the immune system.

Increasing brain size may have negative trade-offs on the immune system for at least two reasons [26]. First, like the brain, the immune system is metabolically demanding [27–29] and requires a large expenditure of an individual's acquired resource pool [30]. Therefore, these organs may compete for energy, nutrients or other limited resources. For example, mice artificially selected for maximal metabolic rate have suppressed innate (though not adaptive) immune function [31]. Second, the brain and the immune system share many signalling molecules and pathways, such as steroid hormones, cytokines, chemokines and major histocompatibility gene expression [32]. However, few studies have investigated selective trade-offs between the brain and the immune system. Support for such a trade-off comes from a study of 108 avian families in which behavioural innovative capabilities (used as proxy for brain size [33]) and the richness of parasitic lice (as proxy for immune capability [34]) were positively correlated [35]. A similar pattern was found in seven species of bats, where parasite species richness correlated positively with brain size [36]. By contrast, parasite species richness (a debatable proxy for immune function) and brain size were unrelated in rodent species [26], while in another study the size of immune defence organs of various birds was positively associated with brain size [37].

Our aim was to compare the immune function of guppies that have been selectively bred for large and small brain size [11] to better elucidate the possible trade-offs between brain size and the immune system. We tested for the effects of brain-size selection on the allograft rejection response in male and female guppies selected for large and small brains. We predicted that large-brained fish would show reduced immune responses (tissue rejection) compared with small-brained fish, though it is impossible to predict which aspects of immune function should be affected by differences in brain size. We did not predict sex differences in graft rejection (based on a previous study that found no sex differences in guppies [38]), but there is almost nothing known about this issue in fish. In mammals, sex-specific differences in immune function are commonly found [39], and though females generally have more robust immune responses than males (females are generally more resistant to parasites but suffer from more autoimmune disease [40]), such findings cannot be extrapolated to all species or other taxa.

## 2. Material and methods

### (a) Directional selection on brain weight

We examined the relationship between brain size and immunological response to scale allografts in laboratory lines of Trinidadian guppies that were artificially selected for large or small relative brain size [11,41]. Briefly, these selection lines were generated using a standard bidirectional artificial selection design that consisted of two replicated treatments (three up-selected lines and three down-selected lines). Since brain size can only be quantified after dissection, we allowed pairs to breed at least two clutches first, then sacrificed the parents for brain quantification and used the offspring from parents with large or small relative brain size as parents for the next generation. More specifically, to select for relative brain size (controlled for body size), we selected on the residuals from the regression of brain size (weight) on body size (length) of both parents. We started with three times 75 pairs (75 pairs per replicate) to create the first three up- and down-selected lines (six lines in total). We summed up the male and female residuals for each pair and used offspring from the top and bottom 25% of these ‘parental residuals’ to form the next-generation parental groups. We then used the offspring of the 30 pairs with the largest residual sums for up-selection and the 30 pairs with the smallest residual sums for down-selection for each following generation. To avoid inbreeding, full siblings were never mated. See Kotrschal *et al*. [11] for full details about the selection experiment. The selection lines differed in relative brain size by 9% in the F_2_ [11] and up to 14% in the F_3_ generation [42], and body size did not differ between the lines [11,43]. All fish were removed from their parental tanks after birth, separated by sex at the first onset of sexual maturation and then kept in single-sex groups with a maximum density of five individuals in 3 l tanks containing 2 cm of gravel with continuously aerated water. We allowed for visual contact between the tanks. The laboratory was maintained at 26°C with a 12 L : 12 D schedule. Fish were fed a diet of flake food and freshly hatched brine shrimp 6 days per week. All measurements were done blindly since only running numbers identified tanks. We used 60 fully grown and mature F_3_ male and female guppies for our assays, balanced over the three replicates, the two brain-size selection regimes and both sexes.

### (b) Allograft rejection

There are several methods commonly used to assess immune function [44], and these techniques measure different aspects of immunity, such as cellular and humoral immune response (to non-replicative antigens). Most immunocompetence assays probe either innate or adaptive immunity, rarely both. Furthermore, most are of limited use for guppies because these fish are too small to obtain blood or other tissue samples without inflicting serious distress on (or even sacrificing) the animal. Scale allografts are a standard technique in the study of fish immune competence [45,46], and they overcome the previously described limitations because they allow the evaluation of both innate and adaptive immunity and have minimal invasiveness. Moreover, allograft rejection has been previously used in guppies to show how immune responses are modulated by dietary carotenoids [38].

To test for innate immunological response, we performed scale allografts according to methods described by Grether *et al*. [38]. We placed individual guppies (sedated with MS-222) in reciprocal pairs on wet gauze next to each other. Every pair consisted of one fish from the large- and one from the small-brained lines, always of the same replicate and same sex. This design excludes potential biases due to genetic relatedness (and matching at major histocompatibility loci) that control rejection responses [47]. Under a stereomicroscope, we then carefully removed a single scale from the dorsal area of each fish and transferred the scales to the so-created empty scale pocket of the other fish (electronic supplementary material, figure S1). Fish were left to recover for 5 min in Petri dishes with fresh water to ensure settlement of the allografts and then returned to their individual home tanks. During the experiment, all fish were kept in individual 10 l tanks with gravel, java moss and a biological filter.

On days 2, 4, 6 and 8 after the allografting, we observed the fish under a stereomicroscope for evidence of healing, swelling and other signs of inflammation, using the criteria described by Cooper [48] and modified by Grether *et al*. [38]. Briefly, the ‘rejection response’ variable is a composite variable incorporating all observable morphological changes for scale allografts in guppies: level 0, slight swelling only; level 1, swelling or melanocytes disrupted; level 2, swelling and melanocytes disrupted; level 3, swelling, melanocytes disrupted and slight cloudiness; level 4, swelling, melanocytes disrupted or partially absent, and cloudiness; 5, swelling, melanocytes absent and strong cloudiness. To assay rejection due to acquired immune responses, we used the same pairs and repeated the allografting as described above 21 days after the first allografting. We used a different scale: two scale-rows caudal to the first allograft. We again scored the rejection response on days 2, 4, 6 and 8 after allografting. Scoring was made ‘blindly’ with regard to the brain-size selection line and replicate because only running numbers identified holding tanks.

### (c) Sample size

Of the 60 experimental animals, one individual lost the allografted scale after day 4 during the first round of allografting, and one fish died before the first allografting. Because one small-brained female died before the second allografting, her large-brained partner fish also could not be allografted. Finally, one fish died after day 6 of the second allografting. Final sample sizes were therefore (first/second allografting) 58/56 complete datasets, 1/1 incomplete datasets and 1/3 missing datasets.

### (d) Data analyses

To analyse our results of both innate and acquired immune rejection, we first used a general linear mixed-effects model (GLMM_1_) with rejection score as dependent variable, brain-size selection regime, sex and allograft set as fixed factors, individual ID and replicate nested in brain-size selection regime as random factors, and day of experiment and day of experiment squared (to account for a nonlinear association [38]) as covariates. To investigate the innate and acquired immune response, we used two separate analogous models (GLMM_2_ and GLMM_3_). To further investigate which days the immune rejection differed between large- and small-brained individuals, we used eight separate, day-specific GLMMs with immune response as dependent variable, brain-size selection regime and sex as fixed factors, and individual ID and replicate nested in brain-size selection regime as random factors. For all models, we used a stepwise model reduction, based on the lowest Akaike's information criterion, and excluded all non-significant interactions (*p* > 0.3 in all cases). Note that the size of the dataset prevented residual distributions from being perfectly normal; however, they were always biased towards more central estimates. All analyses were done in SPSS 22.0.

## 3. Results

**Figure 1.**
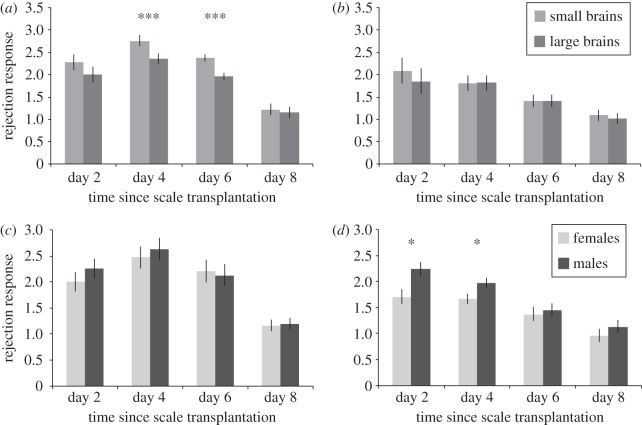
Response to allografted scales by male and female guppies (*Poecilia reticulata*) artificially selected for large and small brain size. The mean response to the first (*a,c*) and second set (*b,d*) of allografts shown in animals from large- and small-brained lines (both sexes combined; *a,b*) and males and females (both brain-size selection lines combined; *c,d*). Bars show the estimated marginal means from GLMMs controlling for replicate and sex (*a,b*), or for replicate and brain-size selection line (*c,d*) (±s.e.), *p < 0.05, ***p ≤ 0.001.

Overall, the first set of allografts, used to assay innate immunity, elicited a stronger rejection response than the second set of allografts, used to measure adaptive immunity; small-brained fish and males tended to show a stronger rejection response than large-brained fish and females; we further found a significant interaction between brain-size selection regime and set of allograft (GLMM_1_: allograft set: *F*_1,394_ = 127.302, *p* < 0.001; sex: *F*_1,56_ = 3.608, *p* = 0.063; brain-size selection regime: *F*_1,56_ = 3.608, *p* = 0.063; day of experiment: *F*_1,395_ = 44.253, *p* < 0.001; day of experiment^2^: *F*_1,395_ = 3.188, *p* = 0.075; allograft set × brain-size selection regime: *F*_1,402_ = 3.932, *p* = 0.048; figure 1).

The first set of allografts elicited a rejection response with a maximum on day 4 (figure 1*a*). In this set, small-brained animals of both sexes showed an overall stronger rejection than large-brained animals (GLMM_2_: sex: *F*_1,55_ = 0.912, *p* = 0.344; brain-size selection regime: *F*_1,55_ = 8.669, *p* = 0.005; day of experiment: *F*_1,169_ = 35.561, *p* < 0.001; day of experiment^2^: *F*_1,169_ = 65.161, *p* < 0.001; figure 1*a*,*c*). When analysing all days separately, we found that on day 4 and day 6 the difference between large- and small-brained animals was significant (figure 1*a*; electronic supplementary material, table S1). In the second set of allografts, the rejection response showed a near-linear decrease over time (figure 1*b*). While brain-size selection regime did not influence this response (figure 1*b*), males mounted a stronger response, and their response declined more steeply over time compared with females (GLMM_3_: sex: *F*_1,177_ = 10.759, *p* = 0.001; brain-size selection regime: *F*_1,4_ = 0.211, *p* = 0.670; day of experiment: *F*_1,167_ = 9.691, *p* = 0.002; day of experiment^2^: *F*_1,167_ = 11.699, *p* = 0.001; sex × day of experiment: *F*_1,167_ = 8.994, *p* = 0.003; figure 1*d*). Visual inspection of figure 1*d* indicates that the faster decrease over time in males compared with females is driven by greater immune response on days 2 and 4.

## 4. Discussion

As predicted, we found that small-brained animals mounted a significantly stronger rejection response to first-set allografts than did large-brained animals. There was no such effect of brain size, however, in the second-set allografts. These results suggest that increased investment into the development of a larger brain leads to a decrease in investment into the innate, but not the adaptive immune system. Our findings therefore support the hypothesis that evolving larger brains has negative trade-offs for immune function, at least for innate immunity.

It is somewhat surprising that the negative effects of increasing brain size on immunity occurred rapidly (within the first 8 days), and presumably via innate responses, since allograft rejection is often assumed to be controlled by adaptive immunity [46,49]. Innate responses to allografts, however, are more important than is generally assumed [50,51].

There are a variety of mechanisms through which selection for brain size can potentially influence immune (innate and adaptive) responses, even directly, since there are several mechanisms that surprisingly control both neurogenesis and immunity [52,53]. For example, toll-like receptors (TLRs) are crucially involved in innate immunity [54], neurogenesis [55] and neurodegeneration [56]. While the transcriptome of adult brains of the brain-size-selected guppies does not differ in TLR expression [57], a detailed investigation of TLRs in several tissues at different developmental stages is needed to elucidate whether changes in TLRs indeed govern a decreased innate immunity when animals evolve larger brains. In addition, the effects we found might be due to changes in the complement system [58], or neutrophils, the first responders to antigen introduction [59,60]. Experiments are therefore planned to investigate characteristics of TLR expression, complement system, neutrophils and other lymphocytes in the large- and small-brained guppies. Comparative analyses are needed to further investigate the relationship between brain size and immune competence, such as white blood cell counts, among different vertebrate species.

Our results might also be due to correlated changes we found in other traits, besides the brain, such as the gut or hormonal system. Those are two non-mutually exclusive explanations of potential indirect pathways by which trade-offs may occur. First, the gut and its microbiome are well-established players in mammalian immune function [61], and recently the role of the gut in the immune defence of fishes has also been emerging [62]. Thus, the fact that in our brain-size-selected guppies the large-brained lines show smaller guts than the small-brained lines [11] may help explain the decreased rejection in large-brained animals. However, whether the size of the gut directly relates to immune competence is currently unknown. Second, we recently found that selection for brain size alters hormonal stress responses, such that large-brained fish secrete less cortisol in a stressful situation compared with small-brained fish [43]. Given that cortisol is immunosuppressive, one would have expected *stronger* immune rejection responses in large-brained fish due to their lower cortisol levels. The fact that we found the opposite for first-set allografts suggests that the effect of the trade-off between brain size and immunity may override the immune-suppressive effects of elevated cortisol levels. Scale autografts, where scales are transplanted between different areas of the same individual, usually do not lead to visible rejection reactions [63]. It is therefore unlikely that bacterial contamination contributed to the observed immune responses. Even if so, our conclusion of decreased innate immune responses in large-brained animals remains unchanged.

So what are the fitness consequences of the negative association we detected between brain size and immune rejection? Does impaired immune function constrain the evolution of larger brains? (We use the term ‘constrain’ in the sense of *impeding* an evolutionary trajectory, as obviously such a trade-off has not *stopped* the evolution of large brains [2,64,65].) Does increasing brain size confer advantages that ameliorate the negative consequences of reduced immune function, such as improved behavioural defences against infectious diseases [66], which could include avoiding contaminated foods [67], rejecting contagious sexual partners [68] and avoiding parasite-infested areas [69]? It is unclear whether such benefits in behavioural strategies could compensate for impairments in innate immunity.

In addition to an apparent trade-off between brain size and immune function, we observed a sex-specific difference in rejection, as females tended to show less pronounced rejection, and especially adaptive immunity was *stronger* in males compared with females. Although the mechanisms underlying a sex-specific innate immune response remains enigmatic, we may speculate on the adaptive value of a decreased adaptive immune response in females. Guppy males use a modified anal fin as intromittent organ to internally fertilize females, and females are frequently exposed to forced, often damaging, copulation attempts [70]. Decreasing the responsiveness of the adaptive immune system may be advantageous for females because the constant development of specific antibodies against each of the large number of males attempting to mate with a female in its lifetime may simply be too costly. If so, the level of coercive mating within a population may drive the responsiveness of the female adaptive immune system. This may help explain the discrepancy between our findings and those of the only other study on scale allografts in guppies, which reported that males and females show equal levels of adaptive immunity [38]. The previous study used animals that were descendants of fish from a low-predation population, where coercive mating is less frequent [71]. Our fish are descendants of animals from a high-predation site [11], where coercive matings are more common. Population-level differences in the level of coercive mating, driven through differences in predation, may therefore underlie variation in female adaptive immune response.

In conclusion, we found evidence for reduced immune response in guppies selected for large brains, which provides support for a functional trade-off between investment into the brain versus immune function. While increased cognitive abilities may ameliorate this trade-off, our findings suggest that immune function is a potential factor constraining the evolution of vertebrate brain size.

## Supplementary Material

Table S1 and Figure S1.

## Data accessibility

Data are deposited in Dryad (doi:10.5061/dryad.7bq5t).

## References

[1] Stearns SC 1992 The evolution of life histories. Oxford, UK: Oxford University Press.

[2] Roff D, Fairbairn D 2007 The evolution of trade-offs: where are we? J. Evol. Biol. 20, 433–447. (10.1111/j.1420-9101.2006.01255.x) 17305809

[3] Charnov EL 1989 Phenotypic evolution under Fisher's fundamental theorem of natural selection. Heredity 62, 113–115. (10.1038/hdy.1989.15) 2732081

[4] Roff DA 1992 The evolution of life histories. New York, NY: Chapman & Hall.

[5] Reznick D, Nunney L, Tessier A 2000 Big houses, big cars, superfleas and the costs of reproduction. Trends Ecol. Evol. 15, 421–425. (10.1016/S0169-5347(00)01941-8) 10998520

[6] Striedter GF 2005 Principles of brain evolution. Sunderland, MA: Sinauer Associates.

[7] Gonda A, Herczeg G, Merila J 2011 Population variation in brain size of nine-spined sticklebacks (*Pungitius pungitius*)—local adaptation or environmentally induced variation? BMC Evol. Biol. 11, 75 (10.1186/1471-2148-11-75) 21435215 PMC3072340

[8] Burns JG, Saravanan A, Rodd FH 2009 Rearing environment affects the brain size of guppies: lab-reared guppies have smaller brains than wild-caught guppies. Ethology 115, 122–133. (10.1111/j.1439-0310.2008.01585.x)

[9] Schillaci MA 2006 Sexual selection and the evolution of brain size in primates. PLoS ONE 1, e62 (10.1371/journal.pone.0000062) 17183693 PMC1762360

[10] Iwaniuk AN, Nelson JE 2001 A comparative analysis of relative brain size in waterfowl (Anseriformes). Brain Behav. Evol. 57, 87–97. (10.1159/000047228) 11435669

[11] Kotrschal A, Rogell B, Bundsen A, Svensson B, Zajitschek S, Brännström I, Immler S, Maklakov AA, Kolm N 2013 Artificial selection on relative brain size in the guppy reveals costs and benefits of evolving a larger brain. Curr. Biol. 23, 168–171. (10.1016/j.cub.2012.11.058) 23290552 PMC3566478

[12] Kotrschal A, Buechel S, Zala S, Corral Lopez A, Penn.J, Kolm N 2015 Brain size affects female but not male survival under predation threat. Ecol. Lett. 18, 646–652. (10.1111/ele.12441). 25960088 PMC4676298

[13] MacLean EL, et al. 2014 The evolution of self-control. Proc. Natl Acad. Sci. USA 111, E2140–E2148. (10.1073/pnas.1323533111) 24753565 PMC4034204

[14] Sol D 2001 The cognitive-buffer hypothesis for the evolution of large brains. In Cognitive ecology II (eds Dukas R, Ratcliff RM), pp. 111–143. Chicago, IL: Chicago University Press.

[15] Kotrschal A, Corral-Lopez A, Zajitschek S, Immler S, Maklakov AA, Kolm N 2015 Positive genetic correlation between brain size and sexual traits in male guppies artificially selected for brain size. J. Evol. Biol. 28, 841–850. (10.1111/jeb.12608) 25705852 PMC4949642

[16] Aiello LC, Wheeler P 1995 The expensive-tissue hypothesis—the brain and the digestive system in human and primate evolution. Curr. Anthropol. 36, 199–221. (10.1086/204350)

[17] Kozlovsky DY, Brown SL, Branch CL, Roth I, Pravosudov VV 2014 Chickadees with bigger brains have smaller digestive tracts: a multipopulation comparison. Brain Behav. Evol. 84, 172–180. (10.1159/000363686) 25059294

[18] Tsuboi M, Husby A, Kotrschal A, Hayward A, Buechel S, Zidar J, Lovle H, Kolm N 2014 Comparative support for the expensive tissue hypothesis: big brains are correlated with smaller gut and greater parental investment in Lake Tanganyika cichlids. Evolution 69, 190–200. (10.1111/evo.12556) 25346264 PMC4312921

[19] Navarrete A, van Schaik CP, Isler K 2011 Energetics and the evolution of human brain size. Nature 480, 91–93. (10.1038/nature10629) 22080949

[20] Tsuboi M, Shoji J, Sogabe A, Ahnesjo I, Kolm N 2015 Testing the expensive tissue hypothesis in a pipefish (*Syngnathus schlegeli*) reveals a negative association between brain size and visceral fat deposits in females. Ecol. Evol. 6, 647–655. (10.1002/ece3.1873). PMC4739565 26865955

[21] Pitnick S, Jones KE, Wilkinson GS 2006 Mating system and brain size in bats. Proc. R. Soc. B 273, 719–724. (10.1098/rspb.2005.3367) PMC1560082 16608692

[22] Speijer D 2012 Brains have a gut feeling about fat storage. Bioessays 34, 275–276. (10.1002/bies.201200002) 22337576

[23] Lemaitre JF, Ramm SA, Barton RA, Stockley P 2009 Sperm competition and brain size evolution in mammals. J. Evol. Biol. 22, 2215–2221. (10.1111/j.1420-9101.2009.01837.x) 20069724

[24] Falconer D.S., Mackay T.F.C 1996 Introduction to quantitative genetics, 4th edn Harlow, UK: Longman.

[25] Agrawal AA, Conner JK, Rasmann S 2010 Tradeoffs and adaptive negative correlations in evolutionary ecology. In Evolution after Darwin: the first 150 years (eds Bell M, Eanes W, Futuyma D, Levinton J), pp. 243–268. Sunderland, MA: Sinauer Associates.

[26] Bordes F, Morand S, Krasnov BR 2011 Does investment into ‘expensive’ tissue compromise anti-parasitic defence? Testes size, brain size and parasite diversity in rodent hosts. Oecologia 165, 7–16. (10.1007/s00442-010-1743-9) 20706848

[27] Lochmiller RL, Deerenberg C 2000 Trade-offs in evolutionary immunology: just what is the cost of immunity? Oikos 88, 87–98. (10.1034/J.1600-0706.2000.880110.X)

[28] Moret Y, Schmid-Hempel P 2000 Survival for immunity: the price of immune system activation for bumblebee workers. Science 290, 1166–1168. (10.1126/science.290.5494.1166) 11073456

[29] Hasselquist D, Nilsson J-Å 2012 Physiological mechanisms mediating costs of immune responses: what can we learn from studies of birds? Anim. Behav. 83, 1303–1312. (10.1016/j.anbehav.2012.03.025)

[30] Råberg L, Nilsson JÅ, Ilmonen P, Stjernman M, Hasselquist D 2000 The cost of an immune response: vaccination reduces parental effort. Ecol. Lett. 3, 382-386. (10.1046/j.1461-0248.2000.00154.x)

[31] Downs CJ, Brown JL, Wone B, Donovan ER, Hunter K, Hayes JP 2013 Selection for increased mass-independent maximal metabolic rate suppresses innate but not adaptive immune function. Proc. R. Soc. B 280, 20122636 (10.1098/rspb.2012.2636) PMC3574324 23303541

[32] Bilbo SD, Schwarz JM 2012 The immune system and developmental programming of brain and behavior. Front. Neuroendocrinol. 33, 267–286. (10.1016/j.yfrne.2012.08.006) 22982535 PMC3484177

[33] Lefebvre L, Reader SM, Sol D 2004 Brains, innovations and evolution in birds and primates. Brain Behav. Evol. 63, 233–246. (10.1159/000076784) 15084816

[34] Schmid-Hempel P, Ebert D 2003 On the evolutionary ecology of specific immune defence. Trends Ecol. Evol. 18, 27–32. (10.1016/S0169-5347(02)00013-7)

[35] Vas Z, Lefebvre L, Johnson KP, Reiczigel J, Rózsa L 2011 Clever birds are lousy: co-variation between avian innovation and the taxonomic richness of their amblyceran lice. Int. J. Parasitol. 41, 1295–1300. (10.1016/j.ijpara.2011.07.011) 21924269

[36] Bordes F, Morand S, Ricardo G 2008 Bat fly species richness in Neotropical bats: correlations with host ecology and host brain. Oecologia 158, 109–116. (10.1007/s00442-008-1115-x) 18679724

[37] Møller AP, Erritzoe J, Garamszegi LZ 2005 Covariation between brain size and immunity in birds: implications for brain size evolution. J. Evol. Biol. 18, 223–237. (10.1111/j.1420-9101.2004.00805.x) 15669979

[38] Grether GF, Kasahara S, Kolluru GR, Cooper EL 2004 Sex-specific effects of carotenoid intake on the immunological response to allografts in guppies (*Poecilia reticulata*). Proc. R. Soc. Lond. B 271, 45–49. (10.1098/Rspb.2003.2526) PMC1691564 15002770

[39] Pennell LM, Galligan CL, Fish EN 2012 Sex affects immunity. J. Autoimmunity 38, J282–J291. (10.1016/j.jaut.2011.11.013) 22225601

[40] Oertelt-Prigione S 2012 The influence of sex and gender on the immune response. Autoimmunity Rev. 11, A479–A485. (10.1016/j.autrev.2011.11.022) 22155201

[41] Kotrschal A, Rogell B, Maklakov AA, Kolm N 2012 Sex-specific plasticity in brain morphology depends on social environment of the guppy, *Poecilia reticulata*. Behav. Ecol. Sociobiol. 66, 1485–1492. (10.1007/s00265-012-1403-7)

[42] Kotrschal A, Corral Lopez A, Amcoff M, Kolm N 2014 A larger brain confers a benefit in a spatial mate search learning task in male guppies. Behav. Ecol. 26, 527–532. (10.1093/beheco/aru227) 25825587 PMC4374130

[43] Kotrschal A et al. 2014 Artificial selection on relative brain size reveals a positive genetic correlation between brain size and proactive personality in the guppy. Evolution 68, 1139–1149. (10.1111/evo.12341) 24359469 PMC4285157

[44] Demas GE, Zysling DA, Beechler BR, Muehlenbein MP, French SS 2011 Beyond phytohaemagglutinin: assessing vertebrate immune function across ecological contexts. J. Anim. Ecol. 80, 710–730. (10.1111/j.1365-2656.2011.01813.x) 21401591

[45] Nevid NJ, Meier AH 1993 A day-night rhythm of immune activity during scale allograft rejection in the gulf killifish, *Fundulus grandis*. Dev. Comp. Immunol. 17, 221–228. (10.1016/0145-305X(93)90041-N) 8325435

[46] Shibasaki Y et al. 2015 Kinetics of lymphocyte subpopulations in allogeneic grafted scales of ginbuna crucian carp. Dev. Comp. Immunol. 52, 75–80. (10.1016/j.dci.2015.04.013) 25917429

[47] Nakanishi T, Fischer U, Dijkstra J, Hasegawa S, Somamoto T, Okamoto N, Ototake M 2002 Cytotoxic T cell function in fish. Dev. Comp. Immunol. 26, 131–139. (10.1016/S0145-305X(01)00055-6) 11696378

[48] Cooper EL 1964 The effects of antibiotics and X-irradiation on the survival of scale homografts in fundulus-heteroclitus. Transplantation 2, 2–20. (10.1097/00007890-196401000-00001) 14113391

[49] Schlitt HJ, Raddatz G, Steinhoff G, Wonigeit K, Pichlmayr R 1993 Passenger lymphocytes in human liver allografts and their potential role after transplantation. Transplantation 56, 951–955. (10.1097/00007890-199310000-00033) 8105572

[50] Fox A, Harrison LC 2000 Innate immunity and graft rejection. Immunol. Rev. 173, 141–147. (10.1034/j.1600-065X.2000.917313.x) 10719675

[51] Land W 2007 Innate immunity-mediated allograft rejection and strategies to prevent it. Transplantation Proceedings 39, 667–672. Amsterdam, The Netherlands: Elsevier. 17445569 10.1016/j.transproceed.2007.01.052

[52] Ziv Y, Avidan H, Pluchino S, Martino G, Schwartz M 2006 Synergy between immune cells and adult neural stem/progenitor cells promotes functional recovery from spinal cord injury. Proc. Natl Acad. Sci. USA 103, 13 174–13 179. (10.1073/pnas.0603747103) PMC1559772 16938843

[53] Ziv Y, Schwartz M 2008 Immune-based regulation of adult neurogenesis: implications for learning and memory. Brain Behav. Immunity 22, 167–176. (10.1016/j.bbi.2007.08.006) 17905567

[54] Takeda K, Akira S 2005 Toll-like receptors in innate immunity. Int. Immunol. 17, 1–14. (10.1093/intimm/dxh186) 15585605

[55] Rolls A, Shechter R, London A, Ziv Y, Ronen A, Levy R, Schwartz M 2007 Toll-like receptors modulate adult hippocampal neurogenesis. Nat. Cell Biol. 9, 1081–1088. (10.1038/ncb1629) 17704767

[56] Okun E, Griffioen KJ, Lathia JD, Tang S-C, Mattson MP, Arumugam TV 2009 Toll-like receptors in neurodegeneration. Brain Res. Rev. 59, 278–292. (10.1016/j.brainresrev.2008.09.001) 18822314 PMC2679904

[57] Chen Y-C, Harrison PW, Kotrschal A, Kolm N, Mank JE, Panula P 2015 Expression change in *Angiopoietin*-1 underlies change in relative brain size in fish. Proc*.* R*.* Soc*.* B 282, 20150872 (10.1098/rspb.2015.0872) PMC4590489 26108626

[58] Stephan AH, Barres BA, Stevens B 2012 The complement system: an unexpected role in synaptic pruning during development and disease. Annu. Rev. Neurosci. 35, 369–389. (10.1146/annurev-neuro-061010-113810) 22715882

[59] Iwama G, Nakanishi T 1996 The fish immune system: organism, pathogen, and environment. New York, NY: Academic Press.

[60] Nathan C 2006 Neutrophils and immunity: challenges and opportunities. Nat. Rev. Immunol. 6, 173–182. (10.1038/nri1785) 16498448

[61] Ley RE et al. 2008 Evolution of mammals and their gut microbes. Science 320, 1647–1651. (10.1126/science.1155725) 18497261 PMC2649005

[62] Rombout JH, Yang G, Kiron V 2014 Adaptive immune responses at mucosal surfaces of teleost fish. Fish Shellfish Immunol. 40, 634–643. (10.1016/j.fsi.2014.08.020) 25150451

[63] Cardwell T, Sheffer R, Hedrick P 2001 MHC variation and tissue transplantation in fish. J. Heredity 92, 305–308. (10.1093/jhered/92.4.305) 11535641

[64] Mezey JG, Houle D 2005 The dimensionality of genetic variation for wing shape in *Drosophila melanogaster*. Evolution 59, 1027–1038. (10.1111/j.0014-3820.2005.tb01041.x) 16136802

[65] Kirkpatrick M, Lofsvold D 1992 Measuring selection and constraint in the evolution of growth. Evolution 46, 954–971. (10.2307/2409749) 28564407

[66] Hart BL 1990 Behavioral adaptations to pathogens and parasites: five strategies. Neurosci. Biobehav. Rev. 14, 273–294. (10.1016/S0149-7634(05)80038-7) 2234607

[67] Karvonen A, Seppälä O, Valtonen E 2004 Parasite resistance and avoidance behaviour in preventing eye fluke infections in fish. Parasitology 129, 159–164. (10.1017/S0031182004005505) 15376775

[68] Rosenqvist G, Johansson K 1995 Male avoidance of parasitized females explained by direct benefits in a pipefish. Anim. Behav. 49, 1039–1045. (10.1006/anbe.1995.0133)

[69] Stanback MT, Dervan AA 2001 Within-season nest-site fidelity in Eastern Bluebirds: disentangling effects of nest success and parasite avoidance. The Auk 118, 743–745. (10.1642/0004-8038(2001)118%5B0743:WSNSFI%5D2.0.CO;2)

[70] Houde A 1997 Sex, color, and mate choice in guppies. Princeton, NJ: Princeton University Press.

[71] Farr JA 1975 The role of predation in the evolution of social behavior of natural populations of the guppy, *Poecilia reticulata* (Pisces: Poeciliidae). Evolution 29, 151–158. (10.2307/2407148) 28563284

[72] [Anon]. 2004 Guidelines for the treatment of animals in behavioural research and teaching. Anim. Behav. 67, i–vi. (10.1006/J.Anbehav.2003.2068) 10640387

