# Supplementary material for: Selection for brain size impairs innate, but not adaptive immune responses
Source: Proc Biol Sci. 2016 Mar 16;283(1826):20152857. doi: 10.1098/rspb.2015.2857 (PMC4810857; doi:10.1098/rspb.2015.2857)
Supplement: Table S1 and Figure S1. [file rspb20152857supp1.docx]

**Electronic Supplementary Material**

for

**Selection for brain size impairs innate, but not adaptive immune responses**

Alexander Kotrschal^1,2^, Niclas Kolm^1^, Dustin J. Penn^2^

^1^ Department of Zoology, Stockholm University, SE-10691, Stockholm, Sweden

^2^ Konrad Lorenz Institute of Ethology, Department of Integrative Biology and Evolution, University of Veterinary Medicine, Vienna, Savoyenstraße 1a, 1160-Vienna, Austria

**Table S1**. Results of rejection responses following a first set of allografts (controlling for replicate) analyzed using day-specific GLMMs.

|  | *d.f.* | *F* | *p* |
| --- | --- | --- | --- |
| **Day 2** |  |  |  |
| Sex | 1/51 | 0.994 | 0.324 |
| Brain size selection | 1/51 | 1.232 | 0.272 |
| **Day 4** |  |  |  |
| Sex | 1/52 | 2.513 | 0.119 |
| Brain size selection | 1/52 | 16.301 | < 0.001 |
| **Day 6** |  |  |  |
| Sex | 1/53 | 0.410 | 0.525 |
| Brain size selection | 1/53 | 12.186 | 0.001 |
| **Day 8** |  |  |  |
| Sex | 1/53 | 0.042 | 0.838 |
| Brain size selection | 1/53 | 0.227 | 0.636 |

**Figure S1**. Photograph of an allografted scale of a guppy showing intermediate-level immunological response. Outlined in red is the single alien scale on the dorsal area of a female, which had been transplanted six d before the photograph was recorded. The immune response score for this individual was rated as level 3 out of 5: “swelling, melanocytes disrupted and slight cloudiness” (see main text for description of all levels).
